# Prevalence and predictive value of electrocardiographic abnormalities in pulmonary hypertension: evidence from the Pan-African Pulmonary Hypertension Cohort (PAPUCO) study

**DOI:** 10.5830/CVJA-2017-020

**Published:** 2017

**Authors:** Balieva Irina, Dzudie Anastase, Thienemann Friedrich, U Sani Mahmoud, Pascal Kengne Andre, Sliwa Karen, Balieva Irina, A Voors Adriaan, Dzudie Anastase, Dzudie Anastase, Sliwa Karen, Thienemann Friedrich, O Mocumbi Ana, Karaye Kamilu, U Sani Mahmoud, S Ogah Okechukwu, Pascal Kengne Andre

**Affiliations:** Hatter Institute for Cardiovascular Research in Africa, SAMRC Cape Heart Centre, IDM, Department of Medicine, Faculty of Health Sciences, University of Cape Town, South Africa; Hatter Institute for Cardiovascular Research in Africa, SAMRC Cape Heart Centre, IDM, Department of Medicine, Faculty of Health Sciences, University of Cape Town, South Africa; Hatter Institute for Cardiovascular Research in Africa, SAMRC Cape Heart Centre, IDM, Department of Medicine, Faculty of Health Sciences, University of Cape Town, South Africa; Hatter Institute for Cardiovascular Research in Africa, SAMRC Cape Heart Centre, IDM, Department of Medicine, Faculty of Health Sciences, University of Cape Town, South Africa; Hatter Institute for Cardiovascular Research in Africa, SAMRC Cape Heart Centre, IDM, Department of Medicine, Faculty of Health Sciences, University of Cape Town, South Africa; Hatter Institute for Cardiovascular Research in Africa, SAMRC Cape Heart Centre, IDM, Department of Medicine, Faculty of Health Sciences, University of Cape Town, South Africa; University of Groningen, Groningen, the Netherlands; University of Groningen, Groningen, the Netherlands; Department of Internal Medicine, Douala General Hospital, Douala, Cameroon; NIH Millennium Fogarty Chronic Disease Leadership Programme; Soweto Cardiovascular Research Heart Unit (SOCRU), Department of Medicine, University of the Witwatersrand, Johannesburg, South Africa; Soweto Cardiovascular Research Heart Unit (SOCRU), Department of Medicine, University of the Witwatersrand, Johannesburg, South Africa; Clinical Infectious Diseases Research Initiative, IDM, University of Cape Town; Integerafrica Research and Development, Cape Town; Wellcome Centre Infectious Diseases Research in Africa, Institue of Infectious Diseases and Molecular Medicine, Cape Town; and Department of Medicine, Groote Schuur Hospital, Faculty of Health Sciences, University of Cape Town, South Africa; Instituto Nacional de Saúde; Faculty of Medicine, EduardoMondlane University, Maputo, Mozambique; Department of Medicine, Bayero University, Kano, Nigeria; Department of Medicine, Bayero University, Kano, Nigeria; Department of Medicine, University College Hospital, Ibadan; Ministry of Health, Umuahia, Nigeria; Non-Communicable Diseases Unit, South African Medical Research Council, Cape Town, South Africa

**Keywords:** pulmonary hypertension, electrocardiogram, sub-Saharan Africa, screening

## Abstract

**Background:**

Pulmonary hypertension (PH) is prevalent in Africa and is still often diagnosed only at an advanced stage, therefore it is associated with poor quality of life and survival rates. In resource-limited settings, we assessed the diagnostic utility of standard 12-lead electrocardiograms (ECG) to detect abnormalities indicating PH.

**Methods:**

Sixty-five patients diagnosed with PH were compared with 285 heart disease-free subjects. The prevalence and diagnostic performance of ECG features indicative of PH and right heart strain were calculated.

**Results:**

Compared to the control group, all abnormalitieswere more frequent in the PH cohort where no patient hada completely normal ECG. The most prevalent (cases vscontrol) ECG abnormalities were: pathological Q wave inat least two contiguous peripheral leads (47.7 vs 6.7%), leftventricular hypertrophy (38.5 vs 9.8%) and p-pulmonale(36.9 vs 20.7%) (all p < 0.05). The sensitivity of ECG criteriafor right heart strain ranged between 6.2 and 47.7%, whilespecificity ranged between 79.3 and 100%. Negative predictivevalue ranged between 81.5 and 88.9% and positive predictivevalue between 25 and 100%. Positive predictive value waslowest (25%) for right bundle branch block and QRS rightaxisdeviation (≥ 100°), and highest (100%) for QRS axis ≥+100° combined with R/S ratio in V1 ≥ 1 or R in V1 > 7 mm.

**Conclusion:**

When present, signs of PH on ECG strongly indicated disease, but a normal ECG cannot rule out disease. ECG patterns focusing on the R and S amplitude in V1 and right-axis deviation had good specificity and negative predictive values for PH, and warrant further investigation with echocardiography.

## Background

**Fig. 1:**
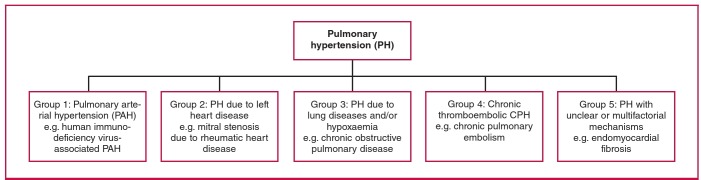
World Health Organisation classification for PH adapted from the 5th World Symposium on Pulmonary Hypertension.^3^

Pulmonary hypertension (PH) is a worldwide public health challenge with an estimated population of affected people in resource-limited countries of 20 to 25 million in 2008.^1^,^2^ Based on shared pathophysiology and disease mechanisms, the World Health Organisation (WHO) and the 5th World Symposium on Pulmonary Hypertension distinguish five groups of PH: arterial (PAH), venous, hypoxic, thromboembolic and miscellaneous^3^ (Fig. 1). Overall, PH results from varying combinations of increases in pulmonary vascular resistance, pulmonary blood flow and pulmonary venous pressure. Sustained pressure overload secondary to chronic PH leads to right ventricular (RV) changes, including hypertrophy, dilatation and RV failure, which are detectable using non-invasive tests such as electrocardiogram (ECG), echocardiography, cardiac magnetic resonance or at best, the gold standard but invasive right heart catheterisation (RHC).

Despite improvements in the understanding of PH and the development of novel therapies, the condition is still diagnosed at an advanced stage in a significant proportion of patients, due to the paucity of symptoms in the early stages of the disease. This has a negative impact on the quality of life and survival rate of patients.^4^ The American College of Cardiology/American Heart Association^5^ and the European Society of Cardiology/European Respiratory Society^4^ guidelines recommend the ECG as an initial tool in diagnosing patients with suspected PH, based on studies done predominantly in patients with PAH. However, these guidelines consider ECG to be an inadequate tool for screening and emphasise the advantage of Doppler echocardiography.

In sub-Saharan Africa (SSA) where chronic and endemic precursors of PH, including chronic infectious diseases, hypertensive heart disease, cardiomyopathy and rheumatic heart disease are highly prevalent,^6^ early diagnosis of PH is of particular relevance. The high cost and low availability of, and need for expertise in echocardiography limit its utility in this part of the world and justify the interest in alternative tests such as ECG.

ECG abnormalities in patients with PH have been predominantly described in other populations.^7^-^11^ The Pan-African Pulmonary Hypertension Cohort (PAPUCO) was established to map out the epidemiology of PH in SSA. In this sub-study, we aimed to assess the predictive value of an affordable, widely available, objective and reproducible test such as ECG to diagnose PH in resource-limited settings.

## Methods

As previously described,^12^ the PAPUCO study was a prospective, registry-type cohort study of PH in Africa. The registry aimed to recruit consecutive patients with newly diagnosed PH based on clinical and echocardiographic criteria, who would be able or likely to return for a six-month follow up, who were at least 18 years old (except for those in paediatric centres in Mozambique and Nigeria), and who consented in writing to participate in the registry.

Centre eligibility included availability of echocardiography, training in assessing right heart function, experience in diagnosing PH according to the WHO classification, experience in clinical management of patients with right heart failure (RHF), and resources to review patients at six-month follow up. Participating centres were invited to join the registry at African cardiac meetings and conferences.^12^ The Heart of Soweto study was a study of 387 urban South Africans of predominantly African descent, determined to be heart disease free (using the Minnesota code) following advanced cardiological assessment, including echocardiography.^13^

PH was diagnosed by specialist cardiologists using the non-invasive definition of PH. The standard is a pathological condition with an increase in mean pulmonary arterial pressure (PAP) beyond 25 mmHg at rest, as assessed by RHC.^14^ Because RHC is seldom available in our setting, PH was diagnosed in patients with a documented elevation in right ventricular systolic pressure (RVSP) above 35 mmHg on transthoracic echocardiography in the absence of pulmonary stenosis and acute RHF, usually accompanied by shortness of breath, fatigue, peripheral oedema and other cardiovascular symptoms, and possibly ECG and chest X-ray changes in keeping with PH, as per the European Society of Cardiology and European Respiratory Society (ESC/ERS) guidelines on PH.^4^

We searched ECGs from the PAPUCO registry to identify all patients who had had both Doppler echocardiography and 12-lead ECG performed within 48 hours of their baseline inclusion. We excluded all patients with pacemakers (due to inapplicability of standard ECG criteria), poor-quality ECGs and those without measurable RVSP. Controls were non-smokers and asymptomatic subjects with normal Doppler echocardiography (and RVSP less than 35 mmHg) who all underwent ECG recordings during their baseline inclusion in the Heart of Soweto study.^15^ This study represents urban South African men and women, all free of any heart disease and other major forms of cardiovascular disease.

All ECGs were reviewed and interpreted by two independent clinical cardiologists who were blinded to the echocardiography results. If consensus could not be reached, a third opinion (AD, FT or KS) was requested. We electively studied pre-specified ECG patterns classified into minor or major abnormalities, as previously described in a large African cohort of heart disease-free Africans.^13^ Minor abnormalities included sinus tachycardia (> 100 beats per min), minor T-wave changes (T-wave flattening) or early repolarisation, definitive right ventricular hypertrophy (QRS axis ≥ +100° or R/S ratio in V1 ≥ 1, or R in V1 > 7 mm or a combination of a right bundle branch block and QRS axis ≥ +100°).

Major abnormalities included:

arrhythmias (supraventricular as premature supraventricular tachycardia, atrial flutter, atrial fibrillation, multifocal atrial tachycardia, paroxysmal atrial tachycardia or ventricular-like premature ventricular complex, ventricular fibrillation, accelerated idioventricular rhythm, Torsades de pointes)
major T-wave abnormalities (T-wave inversion)
left ventricular hypertrophy defined by the Cornell voltage criteria [(S in V3 + R in aVL > 24 mm (men) or > 20 mm (women)]
pathological Q waves
prolonged QTc (> 470 ms as calculated by Bazett’s formula)
left bundle branch block or other conduction delay
p-pulmonale defined as a P wave in lead II > 2 mm or > 1.5 mm in lead V1/V2.

## Statiscal analysis

All statistical analyses were performed with the Statistical Package for the Social Sciences (SPSS) 20.0, Chicago, Illinois. Prevalence, sensitivity (Se), specificity (Sp), and positive (PPV) or negative predictive values (NPV) were calculated by the following formulae:^16^,^17^

Prevalence of an ECG abnormality = total with the abnormality of interest/total number of patients in the group of interest.

Considering echocardiography as our reference diagnostic test in this study (PH present or not), we assessed the diagnostic capability of ECG (ECG criteria positive or negative) in a 2 × 2 contingency table, and calculations were done using the above equation, in which true (false) positive represented our patients (PH group) with (without) ECG abnormalities, while true (false) negative represented controls without (with) ECG abnormalities.

Se = [true positive/(true positive + false negative)] × 100
Sp = [true negative/(true negative + false positive)] × 100
PPV = [sensitivity × prevalence] ÷ [sensitivity × prevalence + (1 – specificity) × (1 – prevalence)]
NPV = [specificity × (1 – prevalence)] ÷ [specificity × (1 – prevalence) + (1 – sensitivity) × prevalence]

Prevalence, Se, Sp, PPV and NPV are presented as percentages,while continuous variables are presented as means and standarddeviation (SD), or median (25th to 75th percentiles). We used χ²to compare proportions of categorical variables and the Student’st-test to compare mean differences for continuous variables. Ap-value < 0.05 was considered statistically significant.

## Results

**Fig. 2:**
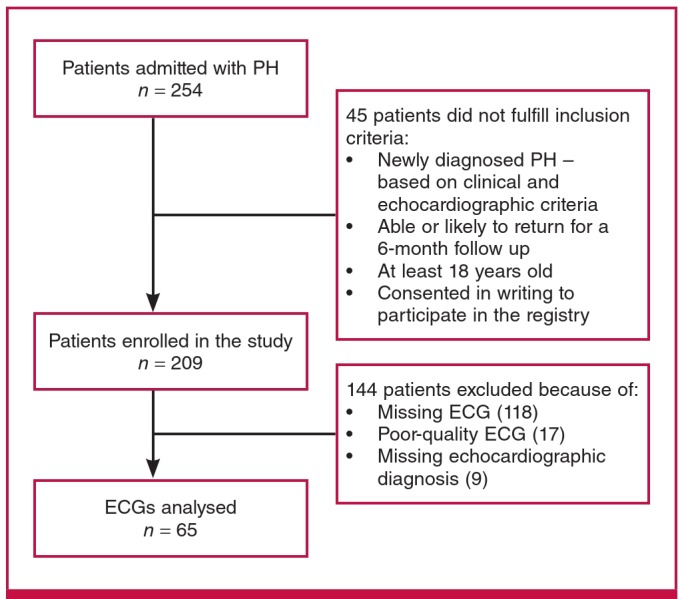
Flow chart of inclusion for the study.

**Fig. 3:**
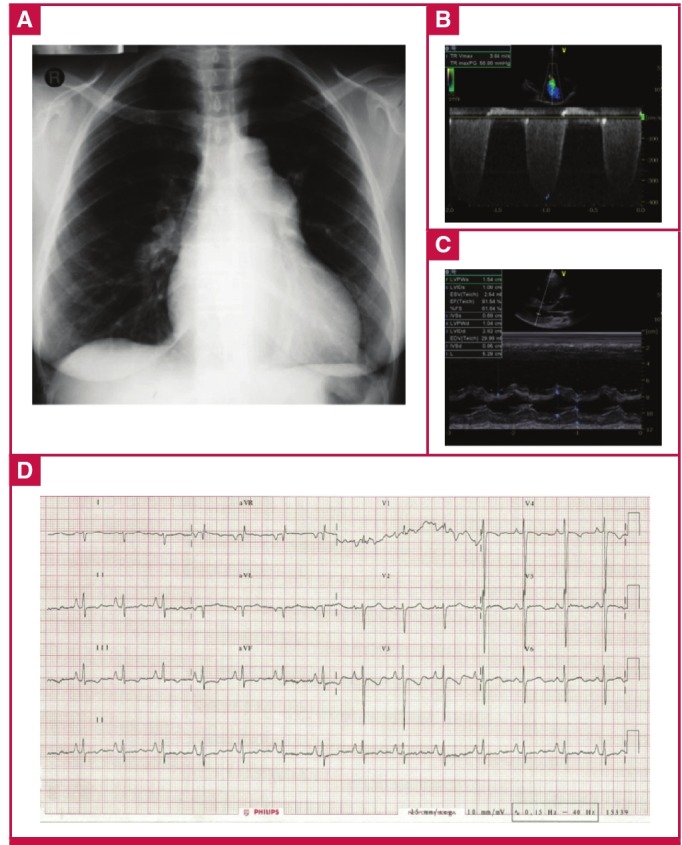
ECG of a 38-year-old HIV-positive woman from the PAPUCO cohort. The patient had been on highly active antiretroviral therapy for three years and presented with palpitations and WHO functional class stage III shortness of breath. The chest X-ray (A) shows mild right heart enlargement and borderline raised cardiothoracic ratio. Doppler echocardiographic images (B, C) confirm the diagnosis of severe PH with both severely enlarged right atrium and ventricle with estimated RVSP of 63 mmHg. The ECG (D) shows a normal heart rate and sinus rhythm, right heart enlargement indicated by right-axis deviation of the QRS complex and by a R/S ratio in lead V1 of > 1 with poor R-wave progression. Right ventricular function was altered with a tricuspid annular plane systolic excursion (TAPSE) of 9 mm. Left ventricular ejection fraction was preserved, there was no valvular heart disease and the pericardium was normal.

Fig. 2 shows how we obtained our cohort of 65 adult patients with ECGs indicating PH from the overall 254 PAPUCO patients. The patients were young (mean age 47 ± 14 years), 21 (32%) were men, and all except four were of black African origin. These four patients were coloured or of mixed race. In Fig. 3, showing a sample ECG, chest X-ray and echocardiographic images of a patient with PH, we describe the clinical context of presentation and confirmation of PH. The control subjects were younger with a mean age of 36 ± 10 years and 48 (16%) were men.

Table 1 summarises the demographic, clinical and echocardiographic profile comparing men and women in the patient group. Significant gender differences were seen, with a higher prevalence of male smokers (47.6 vs 6.8%; p < 0.001) and better performance in the males during the six-minute walking test (352 ± 97 vs 254 ± 142 m, p = 0.017).

**Table 1:** Demographic, clinical and echocardiographic profile of patients with PH in the PAPUCO registry

*Profile*	*All (n = 65) mean ± 2SD,n (%)*	*Male (n = 21) mean ± 2SD,n (%)*	*Female (n = 44) mean ± 2SD, n (%)*	*p-value*
Sociodemographic profile
Mean age (years)	43 ± 15	47 ± 14	41 ± 15	0.133
Smoking				<0.001
Never smoked	44 (67.7)	9 (42.9)	35 (79.5)
Ex-smoker	8 (12.3)	8 (38.1)	0 (0)
Current smoker	5 (7.7)	2 (9.5)	3 (6.8)
Previous or current pulmonary tuberculosis	21 (32.3)	7 (33.3)	13 (29.5)	0.536
Clinical presentation
Dizziness	22 (33.8)	4 (19)	18 (40.9)	0.120
Shortness of breath	56 (86.2)	19 (90.5)	37 (84.1)	0.473
Body mass index (kg/m2)	23.7 ± 5.8	24.1 ± 6.5	23.5 ± 5.5	0.690
Heart rate (bpm)	93 ± 19	91.6 ± 10.2	94.7 ± 5.8	0.195
Pulse oximetry at rest (%)	93.8 ± 7.5	94.7 ± 5.8	91.6 ± 10.2	0.156
Abnormal respiration at rest, n (%)	15 (23.1)	5 (23.8)	10 (22.7)	0.861
Systolic BP (mmHg)	117 ± 22	123 ± 27	114 ± 14	0.186
Diastolic BP (mmHg)	78 ± 16	82 ± 19	77 ± 14	0.238
WHO functional class III or IV	36 (55.4)	13 (61.9)	23 (52.3)	0.323
Karnofsky performance score (%)	67 ± 17	69 ± 15	67 ± 18	0.717
Distance walked in 6-min walking test (m)	280 ± 138	352 ± 97	254 ± 142	0.017
Jugular venous distension	56 (86.2)	19 (90.5)	37 (84.1) 0.591	0.591
Peripheral oedema	40 (61.5)	13 (61.9)	27 (61.4)	0.594
Main echocardiography characteristics	
Right ventricular systolic pressure (mmHg)	61.4 ± 19.8	60.5 ± 24.6	61.8 ± 17.2	0.797
Tricuspid annular plane systolic excursion (mm)	14.9 ± 5	14.7 ± 5.8	15 ± 4.5	0.844
Left ventricular ejection fraction (%)	51.6 ± 20	48 ± 17.5	53.2 ± 21	0.357
Mean left ventricular end-diastolic diameter (mm)	49.6 ± 12.3	52.4 ± 9.9	48.1 ± 13.2	0.210
Right ventricular Enlargement	56 (86.2)	18 (85.7)	38 (86.4)	0.335
Right atrial enlargement	57 (87.7)	19 (90.5)	38 (86.7)	0.830

Data are % or mean ± SD; p-values based on the t-test, chi-squared or Fisher’s exact test as appropriate; statistical significance is based on p < 0.05. BP, blood pressure; WHO, World Health Organisation.

Based on the WHO classification of PH, group 2 (venous PH) was the most prevalent (46%), followed by group 1 (PAH) (31%), group 3 (hypoxic PH) (22%) and group 5 (miscellaneous PH) (3%). In all, 55.4% of patients presented in the WHO functional class III or IV and the mean Karnofsky performance score was 67 ± 17%.

**Fig. 4:**
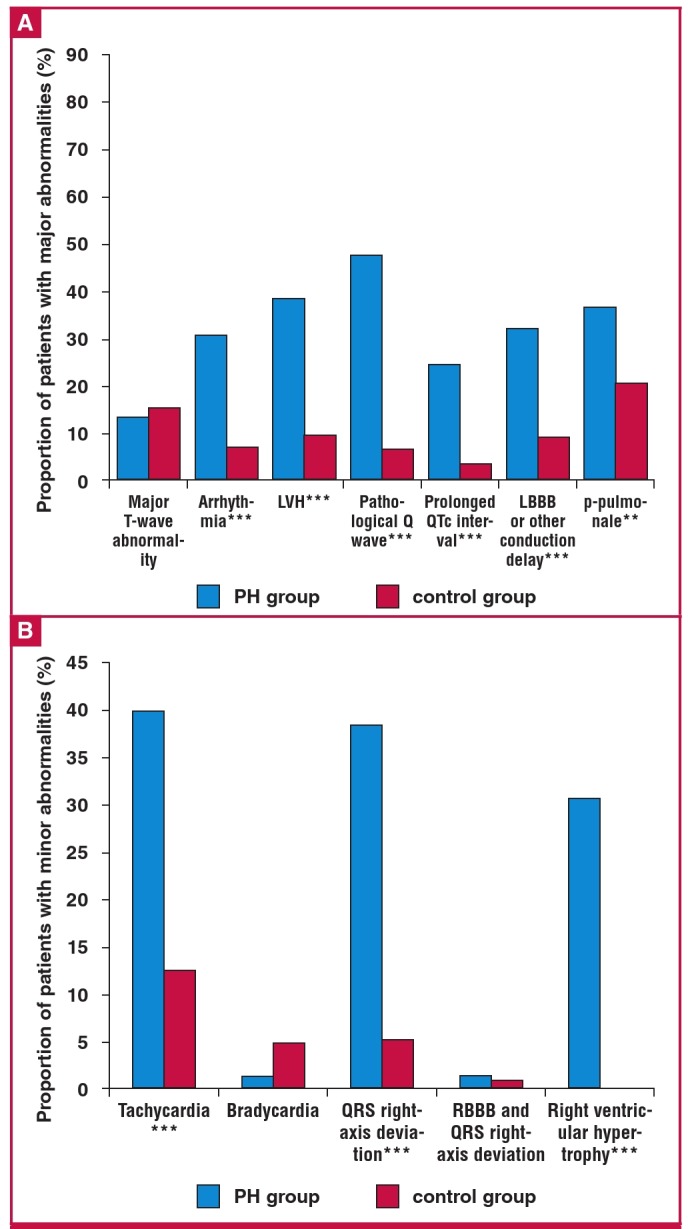
Prevalence of major (A) and minor (B) ECG abnormalities in 65 patients with pulmonary hypertension in the PAPUCO registry compared to 285 controls with normal Doppler echocardiography and right ventricular systolic pressure. RBBB; right bundle branch block, QRS right-axis deviation = QRS axis > 100°. *p < 0.05, **p < 0.01, ***p < 0.001.

Compared to the control group, nearly all abnormalities were much more frequent in our PH cohort. As shown in Fig. 4A, the most prevalent (case vs control) major abnormalities were: pathological Q wave (47.7 vs 6.7%), followed by left ventricular hypertrophy (LVH) (38.5 vs 9.8%) and p-pulmonale (36.9 vs 20.7%). None of the patients had a completely normal ECG, as opposed to 15% in the control group.

Of the minor ECG abnormalities (Fig. 4B), tachycardia (40 vs 12.6%) and QRS axis ≥ 100° (38.5 vs 5.3%) were the most prevalent. In all, 58.5% of the PH group vs 76.5% of the controls were in sinus rhythm. Bradycardia (1.5 vs 4.9%) and right bundle branch block (RBBB) with QRS right-axis deviation (1.5 vs 1.1%) were the least prevalent. Overall, in the PH group, 32.3% had at least three or four major abnormalities and 24.6% had three or four minor abnormalities. The respective numbers in the control group were 4.2 and 0%.

We calculated the predictive values of the ECG patterns suggestive of right ventricular hypertrophy (RVH) or right atrial enlargement (RAE) for the diagnosis of PH. Table 2 shows the sensitivity, specificity and positive and negative predictive values for the occurrence of PH. Sensitivity ranged between 6.2 and 47.7% while specificity ranged between 79.3 and 100%. The NPV ranged between 81.5 and 88.9%. The PPV was lowest at 25% for RBBB and QRS right-axis deviation (≥ 100°), and highest at 100% for QRS axis ≥ 100° combined with R/S ratio in V1 ≥ 1 or R in V1 > 7 mm.

**Table 2:** Predictive values of ECG patterns suggestive of right ventricular hypertrophy or right atrial enlargement for the diagnosis of PH in the PAPUCO registry (RSVP > 35 mmHg)

*ECG criterion*	*Sensitivity (%)*	*Specificity (%)*	*Positive predictive values (%)*	*Negative predictive values (%)*
QRS axis ≥ 100°	38.5	94.7	62.5	87.1
Extreme axis deviation (QRS > 190°)	6.2	100.0	100.0	82.4
R/S ratio in V1 > 1 or R in V1 > 7 mm	47.7	95.8	72.1	88.9
Definite right ventricular hypertrophy	30.8	100.0	100.0	86.4
• QRS axis ≥ +100°; and
• R/S ratio in V1 ≥ 1 or R in V1 > 7 mm
Right bundle branch block and QRS right-axis deviation (≥ 100°)	1.5	99.0	25.0	81.5
P > 2.0 mm in lead II or > 1.5 mm in lead V1/V2, unchanged duration	36.9	79.3	28.9	84.6

We calculated the predictive values of the ECG patterns for the diagnosis of indirect signs of PH (RVH or RAE) in patients with PH. The sensitivity for predicting RVH and RAE were relatively similar for all parameters, ranging from 2.1 to 56.3% and 2.6 to 57.9%, respectively (Table 3). The specificity was higher for both RVH and RAE for all parameters (all > 60%). The PPV was found to be higher for RVH than for RAE, for which all parameters had values above 90%. The NPV was higher for RAE than for RVH, but for both, it was relatively low (all < 50%).

**Table 3:** Predictive values of ECG patterns for the diagnosis of indirect signs of pulmonary hypertension (right ventricular hypertrophy or right atrial enlargement) in patients with pulmonary hypertension from the PAPUCO registry

*ECG criterion*	*Sensitivity (%)*		*Specificity (%)*		*Positive predictive values (%)*		*Negative predictive values (%)*	
	*RVH*	*RAE*	*RVH*	*RAE*	*RVH*	*RAE*	*RVH*	*RAE*
QRS axis ≥ 100°	45.8	47.4	85.7	73.9	91.7	75.0	31.6	46.0
Extreme axis deviation (QRS > 190°)	8.3	7.9	100.0	95.7	100.0	75.0	24.2	38.6
R/S ratio in V1 > 1 or R in V1 > 7 mm	56.3	57.9	78.6	65.2	90.0	73.3	34.4	48.4
Definite right ventricular hypertrophy	37.5	42.1	92.9	87.0	94.7	84.2	30.2	47.6
• QRS axis ≥ +100°; and								
• R/S ratio in V1 ≥ 1 or R in V1 > 7 mm
Right bundle branch block and QRS right-axis deviation (≥ 100°)	2.1	2.6	100.0	100.0	100.0	100.0	23.0	38.3
P > 2 mm in lead II or > 1.5 mm in lead V1/V2, unchanged duration	45.8	39.5	85.7	60.9	91.7	62.5	31.6	37.8

RVH, right ventricular hypertrophy; RAE, right atrial enlargement.

## Discussion

The main findings from this study of the predictive value of ECG abnormalities in patients with PH in SSA are the following. A strictly normal ECG was exceptional, with the most prevalent abnormalities being pathological Q wave, tachycardia, QRS right-axis deviation and left ventricular hypertrophy. The specificity of ECG abnormalities suggestive of PH was generally high, but prevalence of those relating to right heart strain were rather less frequent. Altogether, our findings suggest that on their own, ECG abnormalities cannot discriminate patients who are more likely to be diagnosed with PH via costly and technically demanding examinations, nor can they reliably rule out patients for whom such examinations should be withheld.

Previous studies of heart failure in SSA have reported that a completely normal ECG is very rare in the presence of heart disease.^18^,^19^ In general, the ECG abnormalities have higher specificity than sensitivity. The low sensitivity in our study precludes the ECG from being sufficient for screening without complementary tests, but the ECG is a simple, non-invasive and inexpensive test to perform. It could be implemented in screening protocols as a supplement to physical examination, signs and symptoms, exercise test, chest X-ray and medical history of predisposing factors such as chronic infections, chronic obstructive pulmonary disease and congenital heart disease.

The specificity, as well as the NPV, was high for the parameters indicating PH. Positive findings on an ECG could therefore warrant further investigation with more advanced diagnostics. The most useful parameters seemed to be QRS right-axis deviation of more than 100° and R/S ratio in V1 > 1 or R wave in V1 > 7 mm, especially when both were present.

Overall, the indirect ECG features of PH, namely RVH and RAE, had high specificity and high PPV. Both were higher for RVH than for RAE, possibly explained by the fact that RAE was seen only in more advanced disease. Sensitivity and NPV were lower, and the absence of ECG abnormalities indicating RVH or RAE could not exclude their presence. The sensitivity of indirect ECG features was, however, superior to direct ECG indication of PH, suggesting that a positive ECG can point to RVH or RAE better than to PH directly.

Previous studies have assessed the role of ECG in predicting right ventricular dysfunction but not PH directly, therefore offering less opportunity for comparison with our findings. A study conducted in Canada showed that ECG abnormalities suggestive of RVH were rare in patients with normal RVSP, and had a high positive predictive value.20 Although Henkens et al. showed that ECG-derived ventricular gradient was superior to conventional ECG parameters, QRS right-axis deviation, suggesting chronically increased RV pressure load, was shown to have a sensitivity and specificity of 84 and 96%, respectively.^21^

Increased R/S ratio in V1 or increased R wave in V1 was the best predictor for RVH and RAE, which is in agreement with the results of Nagai et al.,^22^ who found that increased R/S ratio in V1 indicated right ventricular systolic dysfunction. Also Al-Naamani et al.^20^ showed that ECG abnormalities in V1 were superior to abnormalities in V5 and V6, possibly due to lower LV influence in the left precordial leads.

Ahearn et al.^23^ found that an ECG was not sufficient for diagnosing PH, although, from all the parameters, QRS ≥ 100° was the best discriminator and was highly suggestive of RV enlargement. This was, however, a study on PAH in particular, therefore not to be extrapolated without caution to PH of other causes. This emphasises the need to analyse the predictive values of ECG according to the aetiology of PH. In our cohort, left heart disease was the aetiology of PH in 46% of the cases. This could also have influenced the ECG results by possibly concealing mild right ventricular involvement.

## Limitations

Our study has some limitations. First, we acknowledge that the gold standard for diagnosing PH is right heart catheterisation. However it is not always accessible or affordable in our setting and it also has a non-negligible procedure-related mortality rate and serious-events risk, even in an expert’s hands.^24^ Doppler echocardiography is a good alternative as it is safe and non-invasive. Furthermore, the study by Janda et al.^25^ showed a good correlation between right ventricular systolic pressure on echocardiography and pulmonary artery systolic pressure on RHC at baseline.

Second, other useful ECG parameters for our calculations were not recorded. These are R in V1 ≥ 7 mm, R in V5 ≤ 5 mm, R in lead I ≤ 1 mm, S in V1 ≤ 2 mm, R/S in V5 ≤ 1 mm and R in V1 + S in V5 ≥ 10 mm. These have previously been shown to have a good positive predictive value,^20^ and including them in a future study may add to the value of an ECG for screening. Other studies on PH from China and America^26^,^27^ found that prolonged QRS and QTc durations were associated with impaired right ventricular function and the prediction of adverse outcomes in PAH.

Third, the small number of participants with PH most likely affected our capacity to detect significant findings. Lastly, the cardiac disease-free sub-group in this study was an external cohort in whom the echocardiographic assessment ruled out only cardiac disease and PH. This, in turn, could have resulted in differences in case-mix and affected the diagnostic performance of ECG abnormalities for PH.

The strengths of this study include the rigorous approach to ECG interpretation and data analysis, and that the data were derived from the first multicentre study of PH across Africa, where the burden of the condition is increasing.

## Conclusions

ECG abnormalities are common in African patients with PH, but those relating to RV strain specifically are less frequent. When present, ECG features suggestive of PH strongly indicate the disease, but a normal ECG does not rule out disease. The presence of QRS right-axis deviation of ≥ 100° and/or R/S ratio in V1 > 1 or R wave in V1 > 7 mm had good specificity and therefore warrants further investigation with echocardiography. Innovative measures in electrocardiography are required to improve the diagnosis of PH in SSA. This could include studies combining ECG with echocardiography, clinical criteria and cardiac biomarkers to better define the criteria for early diagnosis of PH without exposing patients to unnecessary and costly right heart catheterisation in resource-limited settings.

## References

[1] Gidwani S, Nair A (2014). The burden of pulmonary hypertension in resourcelimited settings. Glob Heart.

[2] Mocumbi AO, Thienemann F, Sliwa K (2015). A global perspective on the epidemiology of pulmonary hypertension. JCan Cardiol.

[3] Simonneau G, Gatzoulis MA, Adatia I, Celermajer D, Denton C, Ghofrani A (2013). clinical classificaUpdatedtion of pulmonary hypertension. J Am Coll Cardiol.

[4] Galie N, Hoeper MM, Humbert M, Torbicki A, Vachiery JL, Barbera JA (2009). Guidelines for the diagnosis and treatment of pulmonary hypertension: the Task Force for the Diagnosis and Treatment of Pulmonary Hypertension of the European Society of Cardiology (ESC) and the European Respiratory Society (ERS), endorsed by the International Society of Heart and Lung Transplantation (ISHLT). Eur Heart J.

[5] McLaughlin VV, Archer SL, Badesch DB, Barst RJ, Farber HW, Lindner JR (2009). ACCF/AHA 2009 expert consensus document on pulmonary hypertension a report of the American College of Cardiology Foundation Task Force on Expert Consensus Documents and the American Heart Association developed in collaboration with the American College of Chest Physicians; American Thoracic Society, Inc.; and the Pulmonary Hypertension Association. J Am Coll Cardiol.

[6] Sliwa K, Wilkinson D, Hansen C, Ntyintyane L, Tibazarwa K, Becker A (2008). Spectrum of heart disease and risk factors in a black urban population in South Africa (the Heart of Soweto Study): a cohort study. Lancet.

[7] Pancholy SB, Palamaner  Subash Shantha G, Patel NK, Boruah P, Nanavaty S, Chandran S (2014). Electrocardiogram-based scoring system for predicting secondary pulmonary hypertension: A cross-sectional study. J Roy Soc Med Cardiovasc Dis.

[8] Lau KC, Frank DB, Hanna BD, Patel AR (2014). Utility of electrocardiogram in the assessment and monitoring of pulmonary hypertension (idiopathic or secondary to pulmonary developmental abnormalities) in patients ≤ 18 years of age. Am J Cardiol.

[9] Scherptong RW, Henkens IR, Kapel GF, Swenne CA, van Kralingen KW, Huisman MV (2012). Diagnosis and mortality prediction in pulmonary hypertension: the value of the electrocardiogram-derived ventricular gradient. J Electrocardiol.

[10] Goncalvesova E, Luknar M, Lesny P (2011). ECG signs of right ventricular hypertrophy may help distinguish pulmonary arterial hypertension and pulmonary hypertension due to left ventricular diastolic dysfunction. Bratisl Lek Listy.

[11] Henkens IR, Scherptong RW, van Kralingen KW, Said SA, Vliegen HW (2008). Pulmonary hypertension: the role of the electrocardiogram. Neth Heart J.

[12] Thienemann F, Dzudie A, Mocumbi AO, Blauwet L, Sani MU, Karaye KM (2014). Rationale and design of the Pan African Pulmonary hyperten sion Cohort (PAPUCO) study: implementing a contemporary registry on pulmonary hypertension in Africa.. Br Med J Open.

[13] Sliwa K, Lee GA, Carrington MJ, Obel P, Okreglicki A, Stewart S (2013). Redefining the ECG in urban South Africans: electrocardiographic findings in heart disease-free Africans. Int J Cardiol.

[14] Lau EMT, Tamura Y, McGoon MD, Sitbon O (2015). The 2015 ESC/ERS Guidelines for the diagnosis and treatment of pulmonary hypertension: a practical chronicle of progress. Eur Respir J.

[15] Stewart S, Wilkinson D, Becker A, Askew D, Ntyintyane L, McMurray JJ (2006). Mapping the emergence of heart disease in a black, urban population in Africa: the Heart of Soweto Study. Int J Cardiol.

[16] Altman DG, Bland JM (1994). Diagnostic tests 2: Predictive values. Br Med J.

[17] Altman DG (1994). Diagnostic tests. 1: Sensitivity and specificity. Br Med J.

[18] Karaye KM, Sani MU (2008). Electrocardiographic abnormalities in patients with heart failure. Cardiovasc J Afr.

[19] Dzudie A, Milo O, Edwards C, Cotter G, Davison BA, Damasceno A (2014). Prognostic significance of ECG abnormalities for mortality risk in acute heart failure: insight from the Sub-Saharan Africa Survey of Heart Failure (THESUS-HF). J Card Fail.

[20] Al-Naamani K, Hijal T, Nguyen V, Andrew S, Nguyen T, Huynh T (2008). Predictive values of the electrocardiogram in diagnosing pulmonary hypertension. Int J Cardiol.

[21] Henkens IR, Mouchaers KT, Vonk-Noordegraaf A, Boonstra A, Swenne CA, Maan AC (2008). Improved ECG detection of presence and severity of right ventricular pressure load validated with cardiac magnetic resonance imaging. Am J Physiol Heart Circ Physiol.

[22] Nagai T, Kohsaka S, Murata M, Okuda S, Anzai T, Fukuda K (2012). Significance of electrocardiographic right ventricular hypertrophy in patients with pulmonary hypertension with or without right ventricular systolic dysfunction. Intern Med.

[23] Ahearn GS, Tapson VF, Rebeiz A, Greenfield JC (2002). Jr. Electrocardiography to define clinical status in primary pulmonary hypertension and pulmonary arterial hypertension secondary to collagen vascular disease. Chest.

[24] Hoeper MM, Lee SH, Voswinckel R, Palazzini M, Jais X, Marinelli A (2006). Complications of right heart catheterization procedures in patients with pulmonary hypertension in experienced centers. J Am Coll Cardiol.

[25] Janda S, Shahidi N, Gin K, Swiston J (2011). Diagnostic accuracy of echocardiography for pulmonary hypertension: a systematic review and metaanalysis. Heart.

[26] Sun PY, Jiang X, Gomberg-Maitland M, Zhao QH, He J, Yuan P (2012). Prolonged QRS duration: a new predictor of adverse outcome in idiopathic pulmonary arterial hypertension. Chest.

[27] Rich JD, Thenappan T, Freed B, Patel AR, Thisted RA, Childers R (2013). QTc prolongation is associated with impaired right ventricular function and predicts mortality in pulmonary hypertension. Int J Cardiol.

